# Liver tissue remodeling following ablation with irreversible electroporation in a porcine model

**DOI:** 10.3389/fvets.2022.1014648

**Published:** 2022-11-04

**Authors:** Eva Monleón, Óscar Lucía, Antonio Güemes, Borja López-Alonso, Dolores Arribas, Héctor Sarnago, Alba Hernaez, José Miguel Burdío, Concepción Junquera

**Affiliations:** ^1^Department of Human Anatomy and Histology, University of Zaragoza, Zaragoza, Spain; ^2^Institute for Health Research Aragón (IIS), Zaragoza, Spain; ^3^Department of Electronic Engineering and Communications, University of Zaragoza, Zaragoza, Spain; ^4^Department of Surgery, University of Zaragoza, Zaragoza, Spain; ^5^Department of General Surgery, Hospital Clínico Universitario Lozano Blesa, Zaragoza, Spain

**Keywords:** irreversible electroporation, liver, hepatic stellate cells, extracellular matrix, regeneration

## Abstract

Irreversible electroporation (IRE) is a method of non-thermal focal tissue ablation characterized by irreversibly permeabilizing the cell membranes while preserving the extracellular matrix. This study aimed to investigate tissue remodeling after IRE in a porcine model, especially focusing on the extracellular matrix and hepatic stellate cells. IRE ablation was performed on 11 female pigs at 2,000 V/cm electric field strength using a versatile high-voltage generator and 3 cm diameter parallel-plate electrodes. The treated lobes were removed during surgery at 1, 3, 7, 14, and 21 days after IRE. Tissue remodeling and regeneration were assessed by histopathology and immunohistochemistry. Throughout the treated area, IRE led to extensive necrosis with intact collagenous structures evident until day 1. From then on, the necrosis progressively diminished while reparative tissue gradually increased. During this process, the reticulin framework and the septal fibrillar collagen remained in the necrotic foci until they were invaded by the reparative tissue. The reparative tissue was characterized by a massive proliferation of myofibroblast-like cells accompanied by a complete disorganization of the extracellular matrix with the disappearance of hepatic architecture. Hepatic stellate cell markers were associated with the proliferation of myofibroblast-like cells and the reorganization of the extracellular matrix. Between 2 and 3 weeks after IRE, the lobular architecture was almost completely regenerated. The events described in the present study show that IRE may be a valid model to study the mechanisms underlying liver regeneration after extensive acute injury.

## Introduction

Irreversible electroporation (IRE) is a method of non-thermal focal tissue ablation, in which short, high-voltage electric pulses are applied to cells to irreversibly permeabilize the cell membranes and consequently induce cell death (1). IRE is being used mainly in tumors where traditional minimal invasive ablation and surgical resection are unavailable. In the case of liver tumors, IRE is particularly valuable in the treatment of patients with tumors adjacent to critical structures, such as the gallbladder, large vessels or bile ducts (2, 3). Furthermore, IRE has also emerged as a promising technique for decellularization of donor organs in the context of organ engineering (3). IRE is characterized by a selective interaction of the electrical fields with the cell membranes only, and no cellular structures, such the extracellular matrix (ECM), are preserved (4). By applying IRE, decellularized tissue scaffolds with native ECM can be created and used for exogenous cells engraftment (5–7) or recellularization after implantation into a recipient (8).

In a healthy liver, the ECM is regulated by quiescent hepatic stellate cells (HSCs) through the secretion of ECM proteins, degrading enzymes and their tissue inhibitors. Quiescent HSCs are liver-resident mesenchymal cells located in the perisinusoidal space of Disse and are characterized by the storage of vitamin A in lipid droplets. In the injured liver, quiescent HSCs transdifferentiate into proliferative, migratory, and contractile activated myofibroblast-like cells (activated HSCs), which secrete abundant ECM proteins that accumulate and form scar tissue (9–11). If the injury persists, liver fibrosis, which is characterized by the net accumulation of extracellular matrix, occurs (12).

In recent years, numerous studies examining the early effects of IRE on liver tissue have been published (7, 13–18), but long-term studies are scarce (4, 18, 19). In addition, although it is well known that IRE treatments preserve extracellular architecture, few studies have focused on the reorganization of liver architecture throughout the regenerative process after IRE (20). The primary aim of this study was to investigate tissue remodeling after IRE in the porcine liver, especially focusing on ECM and HSCs. HSCs express a variety of mesenchymal markers, such as desmin, alpha smooth muscle actin (SMA) and vimentin, and neural markers, such as glial fibrillary acidic protein (GFAP), neurotrophins and synaptophysin. HSC markers have been studied extensively in murine models and in humans, and many discrepancies have been described between them [review by (10, 21)]. In the present study, we used a porcine model, which is a model that has been increasingly used in liver biomedical research because the size, physiology and anatomy of the porcine liver are similar to those of the human liver (22). Although the tissue structure of the human and porcine liver is similar, the typical amount of connective tissue is considerably higher in porcine livers and already resemble fibrosis in human livers (23). Therefore, the secondary aim of this study was to investigate the expression of HSC markers in normal and electroporated porcine livers.

## Materials and methods

The electrodes, generator, animal model, surgical procedure and IRE settings have been previously described in detail (24) and are described in brief in the following sections.

### Animals

Eleven healthy female pigs (Large white × Landrace) weighing 45 kg were used in the present study. Animals were obtained from Inga Food, Nutreco (Zaragoza, Spain). The study was carried out in the facilities of the Institute of Health Sciences (IIS Aragón, Spain), in accordance with the recommendations of the University of Zaragoza for the care and use of experimental animals. The protocol was approved by its Animal Ethics Committee (Permit Number: PI19/16).

### Liver irreversible electroporation

**Figure 1:**
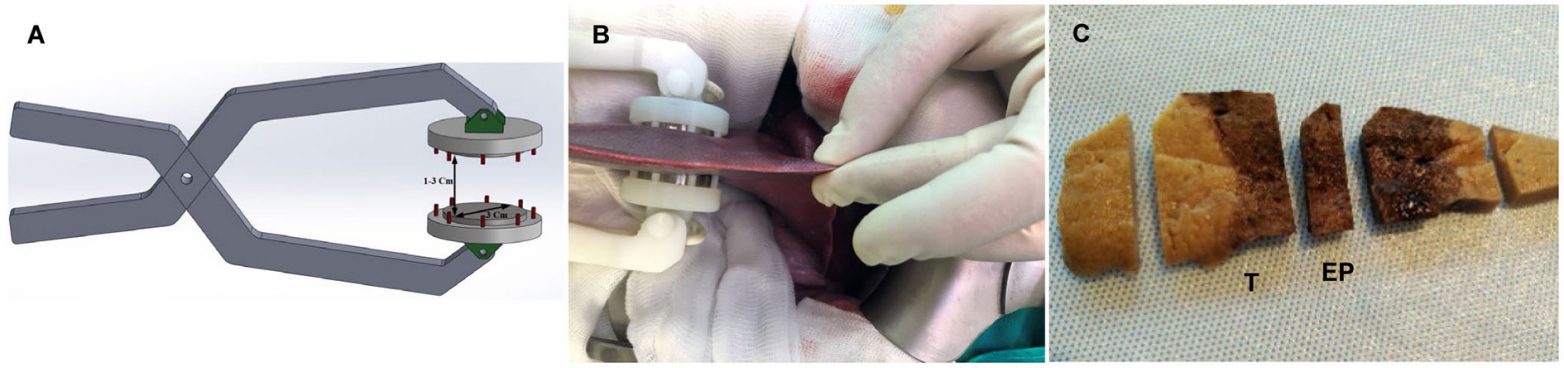
**(A)** Schematic representation of the electrode. **(B)** Picture of the electrode and liver lobe positioning during the procedure. **(C)** Section of fixed porcine liver 1 day after IRE showing a uniform hemorrhagic lesion through the entire ablated zone. (T) sample from the transition zone between treated and non-treated tissue, (EP) sample from the center of the treated zone.

Irreversible electroporation was carried out using a versatile high-voltage generator and parallel-plate electrodes developed by the Group of Power Electronics and Microelectronics (25). The high-voltage generator uses a modular multilevel architecture that enables up to 10 kVpp and 400 App pulses. Digital control using a field-programmable gate array enables versatile implementation to control the pulse and burst number and spacing (26). Three-cm diameter stainless-steel parallel-plate electrodes were used to apply an electric field of 2,000 V/cm using 100 pulses of 100-μs width with a separation of 2 s to avoid thermal effects. The distance between electrodes was 1.5 cm, although it varied slightly between animals and the exact electrodes placement (Figures 1A,B), and the applied voltage was consequently adapted to achieve the desired electric field.

### Sampling

The electroporated lobe was removed during surgery at 1 (D1; *n* = 2), 3 (D3; *n* = 2), 7 (D7; *n* = 3), 14 (D14; *n* = 2), and 21 (D21; *n* = 2) days after IRE. A 0.5-cm thick slice from the center of the electroporated area (including the transition zone between treated and non-treated tissue) was immediately collected and immersed in 10% formalin. After sampling, the animals were euthanized using a potassium chloride injection (1 mEq/kg).

Samples from 5 pigs from an unrelated study were used as controls (Permit number: PI 13/10). In this case, animals were sacrificed using intravenous pentobarbital injections (Dolethal; 10 mg/kg), and liver samples were collected during necropsy.

### Histological analyses

Formalin-fixed liver samples were trimmed perpendicular to the capsule surface, and two samples were obtained: (i) an EP sample, which consisted of a sample from the center of the electroporated zone that only contained treated tissue; and (ii) a T sample, which consisted of a sample from the transition zone between treated and non-treated tissue (Figure 1C).

Samples were processed according to standard histological procedures and stained with hematoxylin and eosin (H&E). Masson's trichrome (MAS) and reticulin staining were performed to evaluate the hepatic connective tissue. Histological processing and staining were carried out by the Scientific Technical Services - Microscopy and Pathology at IIS Aragón.

### Immunohistochemistry analyses

Immunohistochemistry was performed using the following antibodies: monoclonal mouse anti-human actin (SMA), monoclonal mouse anti-human desmin, polyclonal rabbit anti-GFAP and monoclonal mouse anti-human synaptophysin.

Tissue sections (4 μm) were dewaxed in xylene and graded alcohols, hydrated and washed in Tris-buffered saline. Antigen retrieval was performed by heating tissue sections at 96°C for 20 min in EnVision^TM^ FLEX Target Retrieval solution. After antigen retrieval, the following steps interceded by washes with Tris-buffered saline were performed: (1) addition of blocking solution for 5 min to block endogenous peroxidases; (2) incubation for 30 min with primary antibodies; (3) incubation for 30 min with EnVision^TM^ FLEX solution for the purposes of visualization; (4) staining for 10 min with diaminobenzidine, which serves as the chromogen. Finally, the sections were washed in distilled water and counterstained with hematoxylin for 5 min. All incubations were performed at room temperature.

Based on the antibody manufacturer's instructions, porcine colon sections were used as a positive control for all the antibodies. In addition, porcine central nervous system sections were used for GFAP and synaptophysin (Supplementary Figure 1). As a negative control, electroporated and non-electroporated liver samples were subjected to the same procedure in which the primary antibody was replaced with a negative control antibody (Universal Negative Control Mouse or Universal Negative Control Rabbit).

All antibodies and immunohistochemical products were obtained from Dako/Agilent (Santa Clara, CA, United States).

## Results

### Macroscopic evaluation

One day after IRE, a well-demarcated hourglass-shaped uniform hemorrhagic lesion that extended through the entire ablated zone was observed (Figure 1C). On Day 3, a similar lesion was present but with discolored areas in the center of the electroporated zone. On both days, there was an abrupt transition between hemorrhagic lesions and adjacent normal hepatic tissue. By Day 7, the lesion was an extensive zone of pale red discoloration that also had an hourglass shape but with a less clear edge. By Days 14 and 21, the electroporated zone looked like normal hepatic tissue apart from small dark zones in the capsule and subcapsular area that were more evident in one Day 14 case.

### Microscopic evaluation

Apart of controls, all electroporated livers could be classified into 3 groups based on the morphological pattern of the histological and immunohistochemical staining.

**Figure 2:**
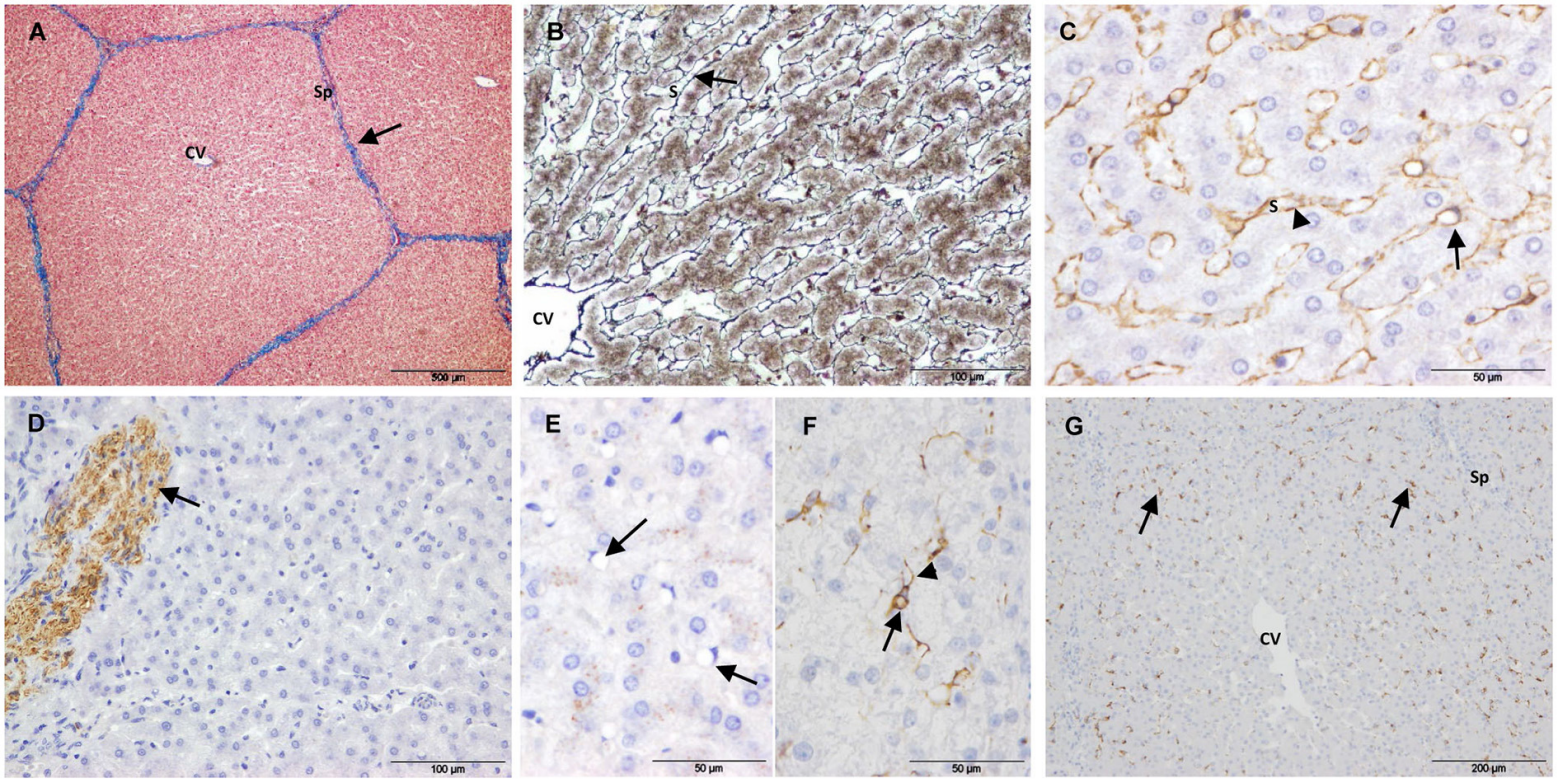
Control samples. **(A–E)** Non-electroporated porcine liver. **(A)** Mason's trichrome stain showing collagen in the portal tracts and septa (arrow). **(B)** Reticulin stain showing reticulin fibers in the space of Disse (arrow). **(C)** SMA immunostaining showing positive HSCs. The arrow points to the body of a positive cell with a large vacuole and a dislocated nucleus. The arrow-heads point to cytoplasmic processes lining the sinusoids. **(D)** GFAP immunostaining showing positive nerve fibers (arrow). **(E)** Desmin immunostaining showing negative HSCs (arrows). **(F,G)** Desmin immunostaining of untreated liver tissue from electoporated liver. **(F)** HSC showing immunostaining in the perinuclear cytoplasm (arrow) extending into cytoplasmic processes (arrow-head). **(G)** Desmin positive HSCs located on the periphery of the lobules (arrows). (CV) Central vein, (Sp) Septum, (S) Sinusoid.

Controls showed the characteristic liver architecture, with the hepatic parenchyma being divided into lobules. MAS staining revealed connective tissue in the septa and portal tracts that surrounded the hepatic lobules (Figure 2A). The hepatocyte trabeculae radiated from a central vein and were separated from adjacent sinusoids by a fine meshwork of reticulin fibers (Figure 2B).

To study quiescent HSC markers, 2 types of control samples were evaluated: i) liver tissue from 5 non-electroporated pigs and ii) non-treated liver tissue from electroporated pigs. Except for desmin immunostaining, the liver tissue was similar in both types of samples. Within the lobules, SMA immunostaining showed a fine layer along the sinusoidal wall throughout the hepatic parenchyma (Figure 2C), and this layer was more intense in the central area of the lobule. Despite strong immunostaining for GFAP and synaptophysin in the enteric and central nervous systems (positive controls, Supplementary Figure 1), no immunostaining was observed associated with HSCs in any case. Occasionally, GFAP (Figure 2D) and synaptophysin were associated with nerve fibers in large septa. In the case of desmin immunostaining, there was a marked variation between controls. In the liver tissue from non-electroporated pigs, most HSCs were negative (Figure 2E), although weakly positive cells were occasionally observed. The *muscularis mucosae* of the colon from these animals (positive control, Supplementary Figure 1) was strongly positive. In the non-treated liver tissue from all electroporated animals, scattered star-shaped desmin-positive cells were observed in the perisinusoidal space, preferentially at the periphery of the lobules (Figures 2F,G).

**Figure 3:**
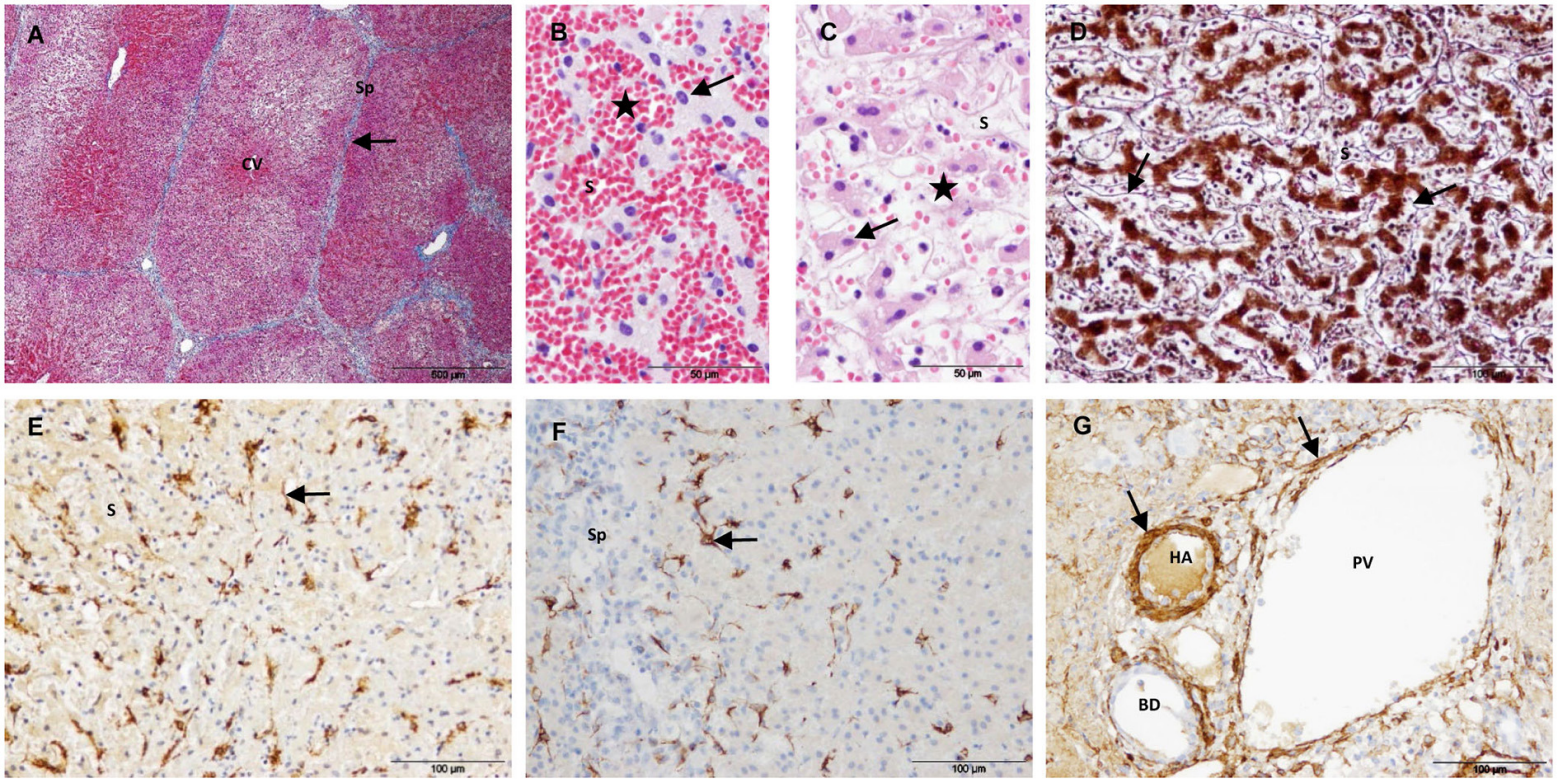
Treated tissue from Group 1 livers (1 day after IRE). **(A)** Mason's trichrome stain showing preserved collagen in the portal tracts and septa (arrow). **(B,C)** H&E stain showing signs of cell death (arrows) and an inhomogeneous congestion of sinusoids (stars). **(D)** Reticulin stain showing preserved reticulin fibers in the space of Disse (arrows). **(E)** SMA immunostaining showing positive HSC with short cytoplasmic processes (arrow). **(F)** Desmin immunostaining showing positive HSC located on the periphery of the lobules (arrow). **(G)** SMA immunostaining showing preserved vascular muscle cells in a portal tract (arrows). (CV) Central vein, (Sp) Septum, (S) Sinusoid, (HA) Hepatic artery, (PV) Portal venule, (BD) Bile duct.

Group 1 included liver samples collected 24 h after IRE. In this group, liver architecture was preserved throughout the sampled tissue, and the treated tissue was characterized by extensive necrosis and hemorrhage. Lobules were divided by fine connective tissue septa, as observed in controls (Figure 3A). Most hepatocytes showed a hypereosinophilic cytoplasm and a pyknotic nucleus. Hemorrhage had an inhomogeneous aspect among the lobules and even within the lobules (Figures 3B,C), suggesting an irregular interruption of blood flow in the treated area. Consistent with this finding, thrombi were observed in some large blood vessels. Within the lobules, hepatic trabeculae were preserved but distorted by the hemorrhage present in sinusoidal spaces. The reticulin meshwork of the sinusoids remained and evidenced a dilated perisinusoidal space with red cell infiltration and signs of edema (Figure 3D). SMA-positive cells were observed along the sinusoids, but the immunostaining pattern was quite different from that seen in the control livers. A stellate morphology was more evident with the presence of thicker and shorter cytoplasmic processes that did not form a continuous layer between hepatic trabeculae and sinusoids (Figure 3E). Star-shaped desmin-positive cells with short processes were also observed; they were usually located on the periphery of the lobules but not on the septa (Figure 3F). In the portal tracts, the structure of bile ducts and vessels appeared preserved, especially in the largest ones. The blood vessel walls showed morphologically intact vascular smooth muscle cells (as revealed by SMA immunostaining) but variable loss of endothelium (Figure 3G).

In the T sample, the lesion showed a hourglass-shape with a well-delineated margin between hemorrhagic necrotic tissue and adjacent normal hepatic tissue.

Group 2 included all liver samples collected 3 and 7 days after IRE and one sample collected at 14 days. In this group, the treated tissue was characterized by the coexistence of areas of necrosis with areas of reparation. In the necrotic tissue, the extracellular structure of the liver appeared preserved. All cases showed a similar pattern of histological changes and immunostaining, but at different degrees of intensity.

**Figure 4:**
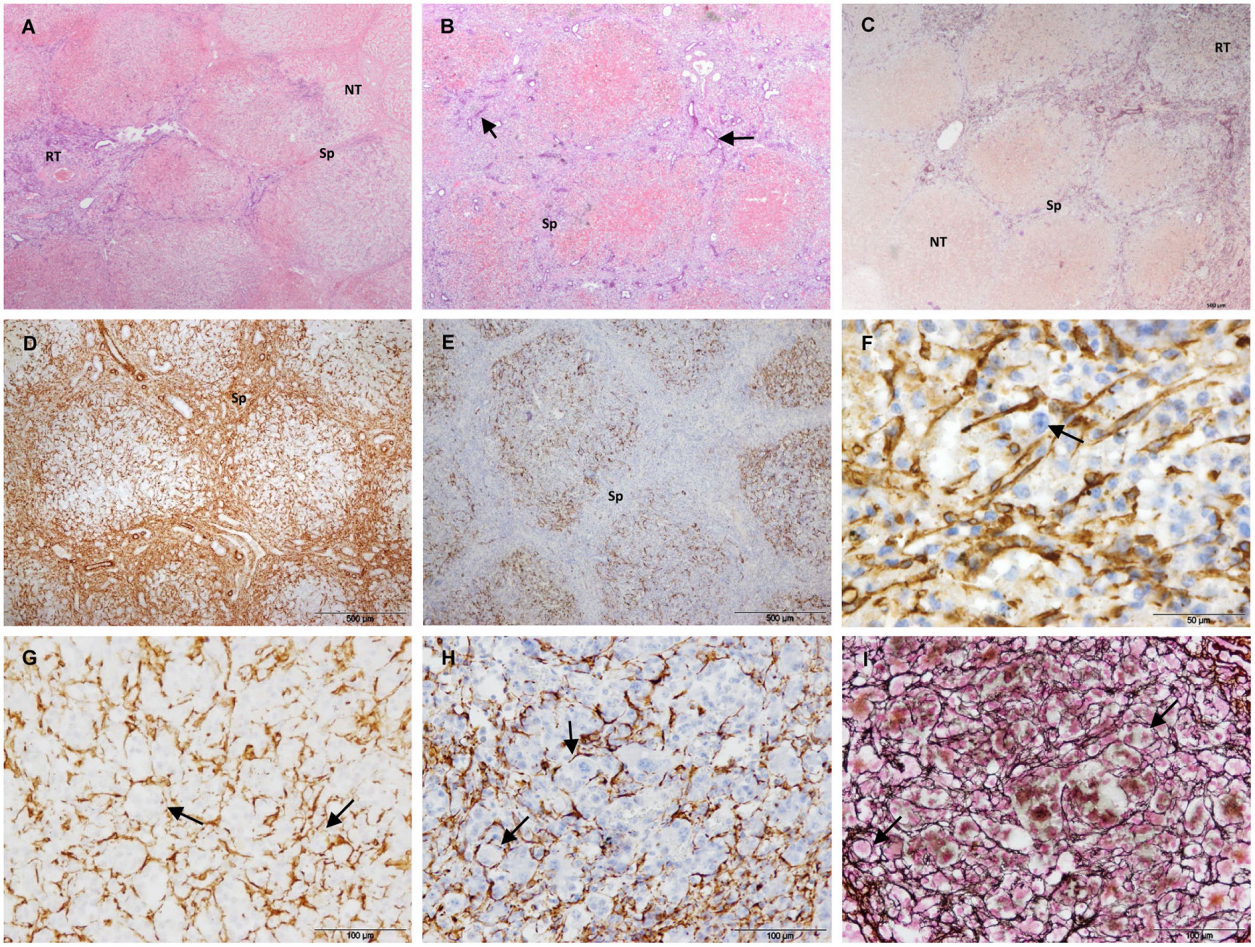
Treated tissue from Group 2 livers (3 days after IRE). **(A)** H&E stain showing the coexistence of an extensive necrosis with reparative tissue. **(B–I)** Histology of the reparative zone. **(B)** H&E stain showing enlarged septa with an intense ductular reaction (arrows). **(C)** SMA immunostaining. In the limit with the necrotic zone positive cells seemed to move toward this zone through preserved septa. **(D)** SMA immunostaining showing an intense immunolabeling in the enlarged septa and within the lobules. **(E)** Desmin immunostaining showing almost no immunolabeling in the enlarged septa but positive cells within the lobules. **(F)** SMA immunostaining showing elongated positive cells within a lobule. Negative round cells can be also observed (arrow). Within the lobules, **(G)** SMA- and **(H)** desmin immunostaining, and **(I)** reticulin patterns were similar and appeared to outline individual cells and round structures (arrows). (Sp) Septum, (NT) necrotic tissue, (RT) reparative tissue.

Three days after IRE, necrosis was an extensive area demarcated by a rim of acute inflammation, hyperemia and hemorrhage (Figure 4A). The reparative zone was characterized by enlarged septa with an evident increase in the connective tissue and vessels, numerous spindle cells and an intense ductular reaction (Figure 4B). In the limit with the necrotic zone, numerous elongated SMA-positive cells seemed to move toward this zone through preserved septa (Figure 4C). In the reparative zone, enlarged septa showed strong SMA immunostaining (Figure 4D) but were almost negative for desmin (Figure 4E). Within the lobules, it was observed a heterogeneous cell population with elongated and large round cells that emanated from the septa and tended to form round structures (Figures 4F–H). SMA- and desmin-positive immunostaining patterns were similar and appeared to outline individual cells and round structures (Figures 4G,H). These immunostaining patterns were found to be correlated with reticulin staining, which changed from the normal trabecular pattern to a round disorganized pattern (Figure 4I).

**Figure 5:**
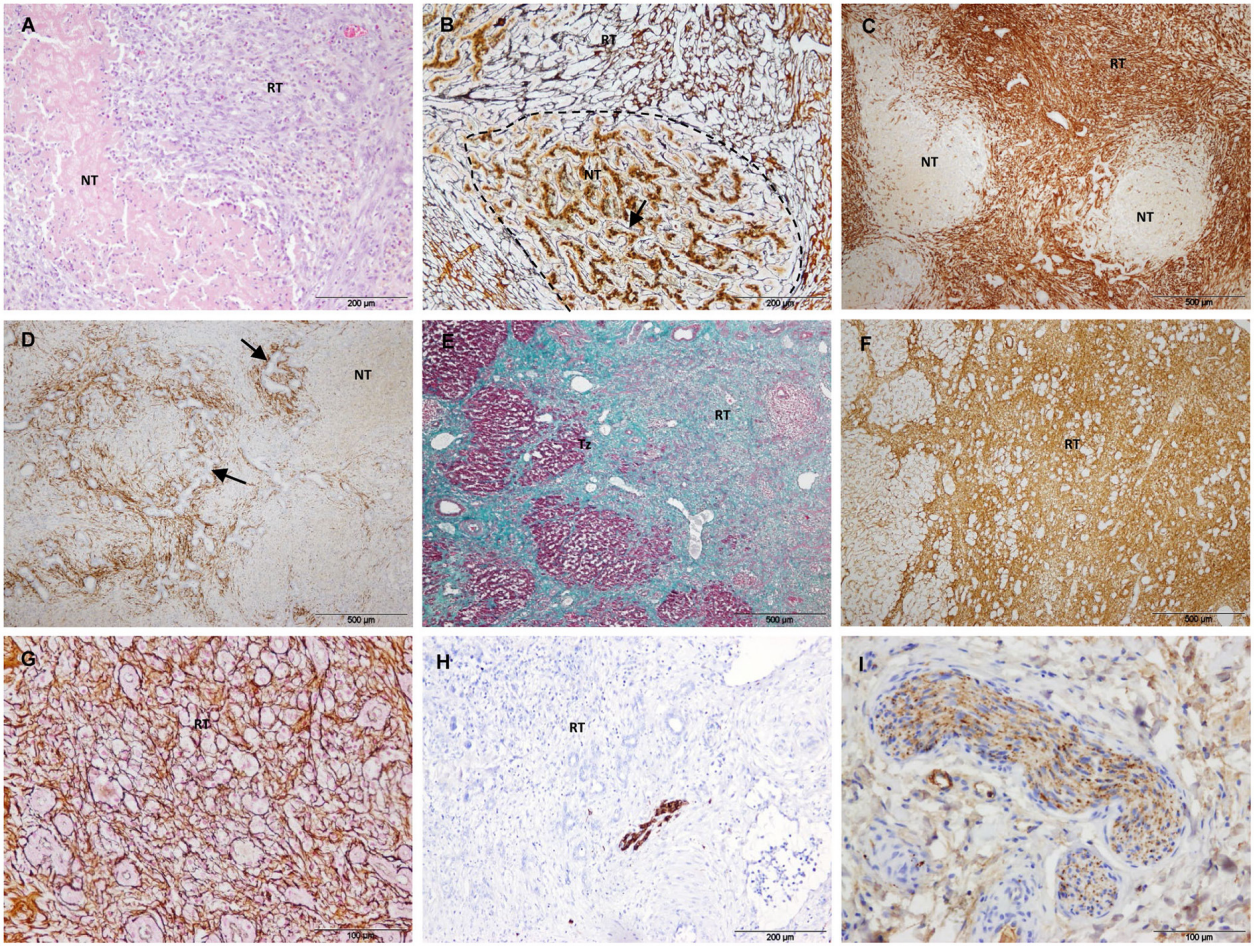
Treated tissue from Group 2 livers (7/14 days after IRE). **(A–D)** Histology of the core of the electroporated sample. **(A)** H&E stain showing a small focus of necrosis surrounded by reparative tissue. **(B)** Reticulin stain showing preserved reticulin fibers (arrow) with a trabecular pattern in a focus of necrosis (delineated section) and a disorganized pattern in the surrounding reparative tissue. **(C)** SMA immunostaining showing foci of necrosis surrounded by intense immunolabeled reparative tissue. **(D)** Desmin immunostaining showing desmin immunostaining in the septa around necrotic foci associated with ductular proliferative reaction (arrows). **(E–G)** Histology of the transition sample. **(E)** Mason's trichrome stain showing a massive increase in collagen in the reparative tissue. **(F)** SMA immunostaining showing an intense immunostaining in the reparative tissue. **(G)** Reticulin stain showing a complete disorganization of the normal trabecular reticulin pattern in the reparative tissue. **(H)** GFAP and **(I)** Synaptophisin immunostaining showing positive nerve fibers in the reparative tissue. (NT) necrotic tissue, (RT) reparative tissue.

After 7/14 days, the necrotic area decreased but foci of necrosis of variable sizes persisted in the core of the electroporated samples (Figures 5A–D). The foci of necrosis maintained the trabecular reticulin pattern (Figure 5B) and were surrounded by reparative tissue with a disorganization of the reticulin pattern, an intense SMA immunostaining and a marked ductular reaction (Figures 5B–D). To a lesser extent, desmin immunostaining was also observed in the septa and was frequently associated with the ductular proliferative reaction (Figure 5D). In the transition sample, a massive increase in collagen and SMA immunostaining with a complete disorganization of the reticulin pattern was observed (Figures 5E–G). In this zone, the lobular architecture of the liver was difficult to distinguish. It should be noted that the D14 sample included in this group had a large septum along the electroporated tissue.

In Group 2, GFAP and synaptophysin immunostaining was observed to be associated with nerve fibers in the reparative tissue (Figures 5H,I) and in non-treated tissue. No immunostaining was observed associated with myofibroblast-like cells.

**Figure 6:**
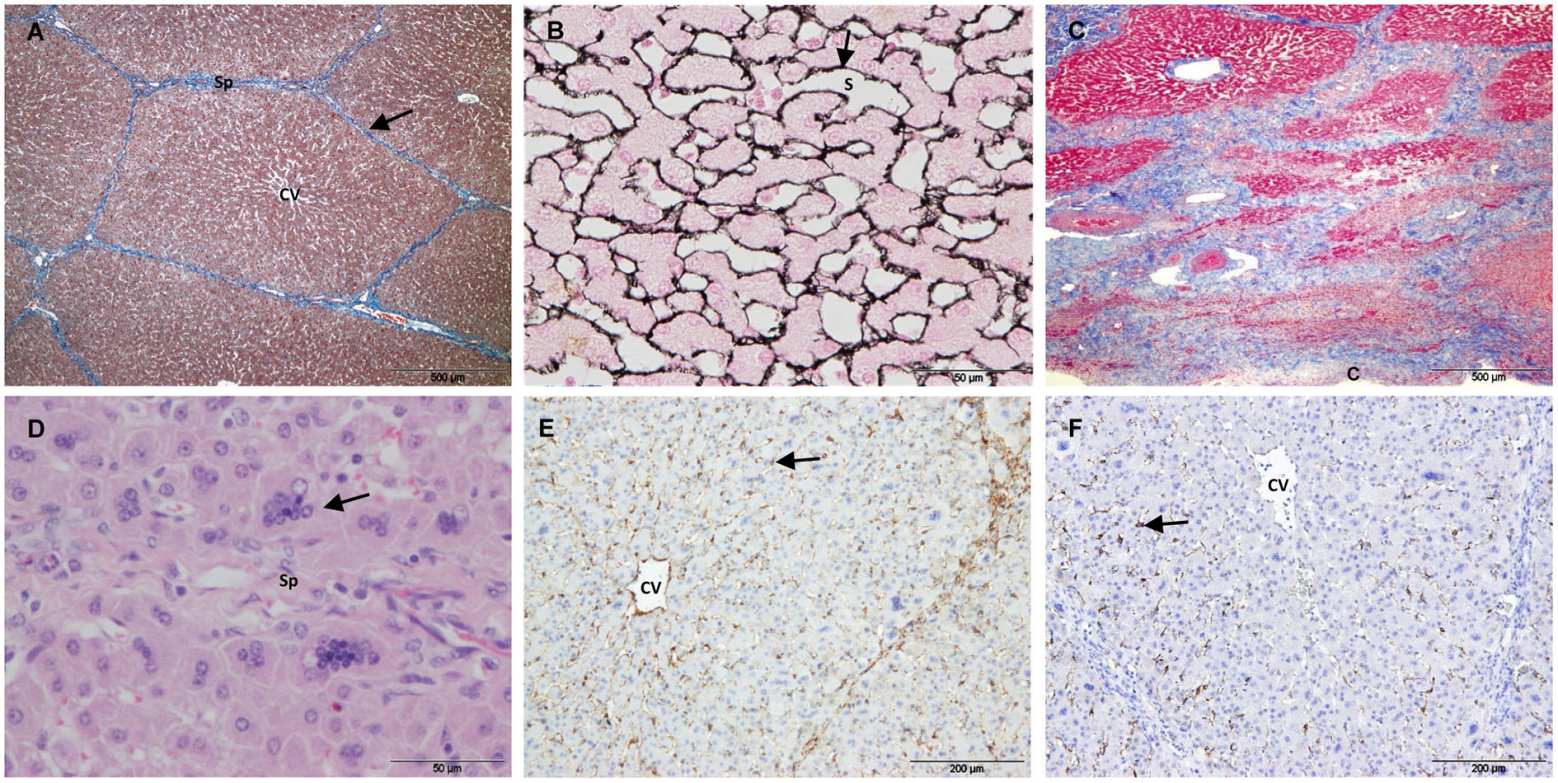
Treated tissue from Group 3 livers (14/21 days after IRE). **(A)** Mason's trichrome and **(B)** reticulin stain showing normal liver architecture, with collagen in the portal tracts and septa (arrow) and reticulin fibers in the space of Disse (arrow). **(C)** Mason's trichrome showing subcapsular fibrosis. **(D)** H&E stain showing multinucleated hepatocytes (arrow). **(E)** SMA immunostaining showing positive HSCs within the lobules (arrow). **(F)** Desmin positive HSCs located on the periphery of the lobules (arrow). (CV) Central vein, (Sp) Septum, (S) Sinusoid, (C) Capsule.

Group 3 included the liver samples collected 21 days after IRE and one sample collected at 14 days. In this group, most of the treated area was regenerated. The lobular architecture and extracellular matrix were similar to those of the control (Figures 6A,B), except for different degrees of subcapsular fibrosis (Figure 6C). In one D21 case, there was increased fibrous tissue near large septa located in the center of the electroporated sample. H&E staining showed dilated centrilobular sinusoids and multinucleated hepatocytes at the periphery of the lobules (Figure 6D). SMA (Figure 6E), GFAP and synaptophysin immunostaining was similar to the control, and desmin immunostaining associated with HSCs was observed at the periphery of the lobules throughout the sample tissue (in treated and non-treated tissue; Figure 6F).

## Discussion

A characteristic that differentiates IRE from other ablation methods is that it preserves collagenous structures and thereby tissue scaffolding (4, 27, 28), which results in a more rapid restoration of tissue perfusion (28). In the present study, we assessed the effects of IRE on the normal porcine liver and the subsequent ECM remodeling process over 3 weeks. Under the conditions of the present study, liver tissue was repaired and almost completely regenerated between 2 and 3 weeks after IRE. We applied supraclinical electroporation settings (29) using 3-cm diameter parallel-plate electrodes, which produced a uniform area of necrosis in the entire treated zone. Current IRE treatments apply needle-type electrodes to perform minimally invasive surgery (30, 31). However, this technique leads to non-homogeneous electric field distribution that can induce white coagulation around the electrodes (14, 16, 18, 32). To study liver reparation after IRE, we considered parallel-plate electrodes that allow the application of a homogeneous electric field between the electrode plates.

Although a distinctive property of IRE is that tissue scaffolding is maintained, to our knowledge, this is the first study on the effect of IRE on the hepatic reticulin network. Reticulin fibers are components of the ECM and consist predominantly of type III collagen. They are located in the space of Disse and form a loose subendothelial matrix that provides stromal support and allow exchange between hepatocytes and sinusoidal blood (33, 34). In the present study we show that the reticulin network remains in the small foci of necrosis that are present up to 7 and 14 days after IRE. These results suggest that the fibrillar collagen within the lobules remains in the necrotic tissue throughout the repair process and may be one of the causes of the rapid hepatic tissue regeneration observed in electroporated animals (29). Previous studies have investigated ECM changes in hepatic regenerative response after IRE using MAS (7, 20). This stain primarily demonstrates type I collagen, which is found in healthy liver in the septa and portal ducts (34).

The reparative process after IRE is represented by samples included in Group 2. This process was characterized by a massive proliferation of myofibroblast-like cells that invaded the necrotic tissue, accompanied by a complete remodeling of the ECM. A large increase in SMA and desmin expression was associated with the myofibroblast-like cell population. The morphological and immunstaining patterns observed suggest that myofibroblasts migrate from the periphery of the lesion toward the necrotic areas through the residual septa and then from the septa toward the center of the necrotic lobules. As the myofibroblasts migrate, they synthesize collagen, which is evidenced by the thickening of the septa and the change in the reticulin pattern. The new reticulin pattern showed a clear association with the desmin and SMA immunopatterns. However, differences were observed in the enlarged septa (type I collagen), which were practically devoid of desmin but stained intensely for SMA. These differences in protein expression suggest the existence of different populations of myofibroblasts in the process of liver regeneration after acute injury. Although HSCs are the main source of collagen-producing myofibroblasts in the injured liver (9, 35–37), we cannot determine whether this finding is due to HSC heterogeneity (37–40) or to the participation of other liver fibroblasts, such as portal fibroblasts (41).

As time went by, a scar that affected almost the entire treated area was observed in Group 2. This scar was characterized by a complete disorganization of the ECM scaffolding with a disappearance of lobular hepatic architecture, a marked proliferation of SMA-expressing myofibroblasts, and a large accumulation of type I collagen. The repair process observed here is consistent with that previously described in a rat model (20). In that model, repair zones with ECM deposition and a heterogeneous cell population including elongated SMA-positive cells are observed as early as 3 days post-IRE. Here we also show that the regenerative process is characterized by a complete reorganization of the reticulin framework. In addition, we describe differences in the protein expression of myofibroblasts in the liver regeneration process after IRE. The events described in these studies perfectly fit the well-known process of hepatic regeneration after an acute insult, in which a temporary scar that provides a provisional matrix for epithelial regeneration is produced at the site of injury (11).

In our study, all treated animals showed a rather similar progression until 7 days after IRE, but the time interval differed in the resolution phase. Whereas, 1 of the 2 cases was still in the reparative process (Group 2) 2 weeks after IRE, the other case was almost completely regenerated (Group 3). Three weeks after IRE, the treated liver tissue of both animals was similar to controls, suggesting that the scarring resolved 2 to 3 weeks after IRE. The presence of large vascular structures and bile ducts and/or thrombi within the EP tissue may explain the differences in the timing of the regeneration process. The presence of these structures has been proposed as a factor that contributes to the heterogeneity in the shape and extension of IRE-induced lesions (17) as well as in the distribution of the electric field (42). Previous reports on the long-term histopathology of IRE in the normal porcine liver also describe discrepancies in this process. While a study described normal liver histology 2 weeks after IRE (19), another study reported that a fibrous scar was observed in the treated area at the same time point (4).

In a healthy liver, the ECM is regulated by quiescent HSCs which transdifferentiate into myofibroblasts in response to a hepatic insult (9, 35–37). HSCs express a variety of markers that have been studied extensively in murine models and in humans, but studies on porcine liver are scarce (10, 21). In the present study, we show that the protein expression profile of porcine quiescent HSCs is different from that described in the literature for mice and humans but similar to that of dogs (10, 21, 43). SMA is classically considered a marker of activated HSCs in human and rodent livers because it is absent from quiescent HSCs (10, 21, 44, 45). In contrast, our results show that SMA is a valid phenotypic marker for quiescent HSC in the porcine model. The discrepancy in results between species could be explained by differences in immunostaining conditions (46). It is also possible that the presence of SMA immunostaining in porcine quiescent HSCs represents an increase in contractility with respect to human or rat cells (43). Desmin is used as a gold standard to identify HSCs in normal and injured rodent livers, but studies on human livers are not consistent (10, 21, 44, 45). In the normal porcine liver, we observed a marked variation in desmin immunostaining between the 2 types of controls used. This might be due to the intrinsic tissue variability regarding sampling (postmortem in non-electroporated pigs), time of fixation and age of the paraffin blocks (longer in non-electroporated pigs), and the age and breed variation of the animals (43). This variation could also be explained by a partial activation of a population of HSCs in the nontreated tissue from electroporated-livers as a response to IRE (39). Synaptophysin and GFAP expression has been described in human and murine HSCs, and GFAP has been proposed as a marker of early activation of human HSCs (47, 48). In our study, HSCs in control and treated tissues were consistently negative for synaptophysin and GFAP as reported in the normal canine liver (43). This result indicates that synaptophysin and GFAP are not valid markers for HSCs in the porcine model. The pig liver is preferably used as a large animal model of acute liver disease in experimental medicine (22). Here we show specie-related differences in the expression of porcine HSC markers that may be taken into account when using the porcine model for liver studies.

## Data availability statement

The raw data supporting the conclusions of this article will be made available by the authors, without undue reservation.

## Ethics statement

The animal study was reviewed and approved by Comisión Ética Asesora para la Experimentación Animal de la Universidad de Zaragoza.

## Author contributions

EM, CJ, ÓL, and AG conceived and designed the experiments. ÓL, JB, HS, and BL-A developed the high-voltage generator and electrodes and performed the IRE. AG, DA, and AH performed surgical processes and the IRE. EM and CJ performed the histological and histopathological analysis and the microscopic evaluation. Acquisition of the financial support for the project and project administration by ÓL, AG, and JB. EM wrote the main manuscript text and prepared figures. All authors read and approved the final paper.

## Funding

This work was supported by the Gobierno de Aragón co-funded by the EU through FEDER (Project LMP106_18), partially supported by the Ministerio de Ciencia e Innovación and the Agencia Estatal de Investigación under Project PID2019-103939RB-I00 co-funded by the EU through FEDER, and by the Instituto de Salud Carlos III under Project PI21/00440.

## Conflict of interest

The authors declare that the research was conducted in the absence of any commercial or financial relationships that could be construed as a potential conflict of interest.

## Publisher's note

All claims expressed in this article are solely those of the authors and do not necessarily represent those of their affiliated organizations, or those of the publisher, the editors and the reviewers. Any product that may be evaluated in this article, or claim that may be made by its manufacturer, is not guaranteed or endorsed by the publisher.
