# Supplementary material for: Liver tissue remodeling following ablation with irreversible electroporation in a porcine model
Source: Front Vet Sci. 2022 Nov 4;9:1014648. doi: 10.3389/fvets.2022.1014648 (PMC9672843; doi:10.3389/fvets.2022.1014648)
Supplement: Supplementary file 1 [file Data_Sheet_1.PDF]

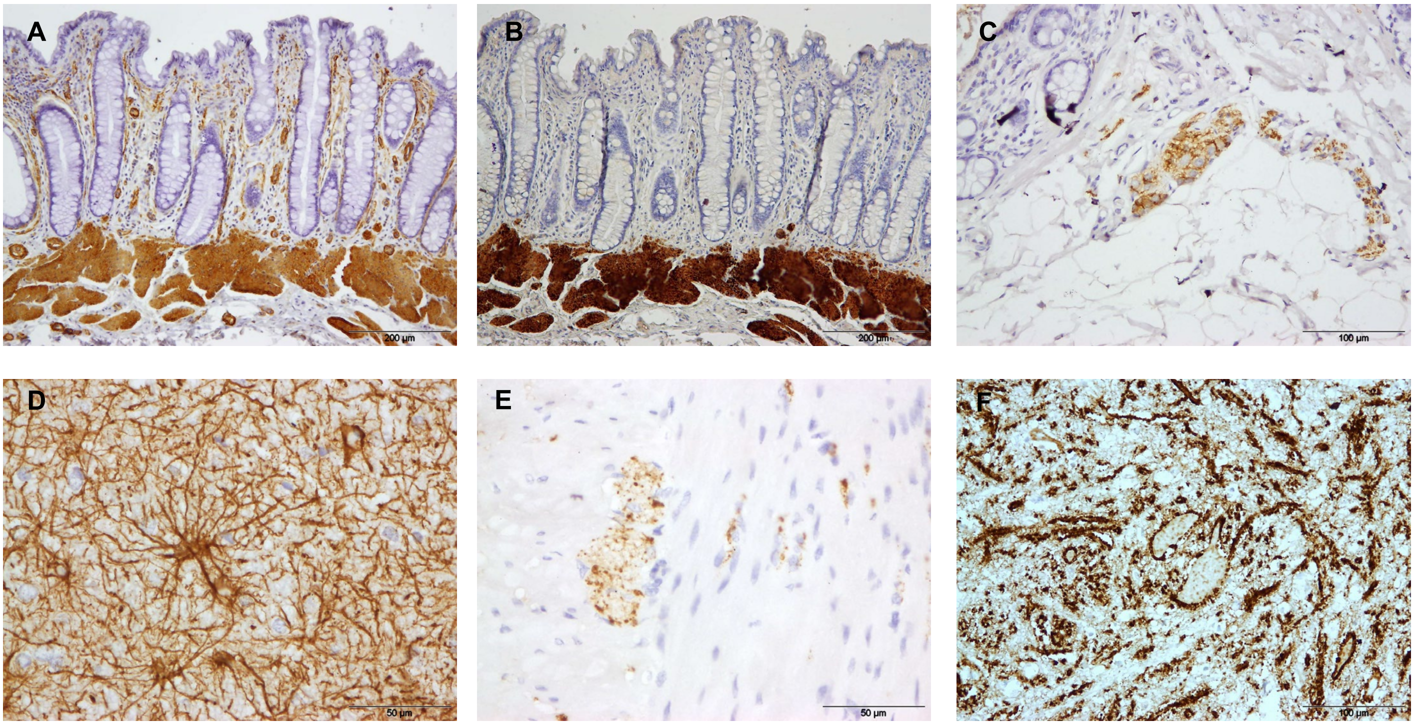

Supplementary Fig. 1. Positive control porcine tissues for immunohistochemistry. Strong immunostaining for SMA (A) and desmin (B) of the *Muscularis mucosae* of the colon. Strong immunostaining for GFAP (C, D) and synaptophysin (E,F) of the enteric and central nervous system.
